# Lawsuit and Traumatic Brain Injury: The Relationship Between Long-Lasting Sequelae and Financial Compensation in Litigants. Results From the PariS-TBI Study

**DOI:** 10.3389/fneur.2019.00320

**Published:** 2019-04-12

**Authors:** Eléonore Bayen, Alexis Ruet, Claire Jourdan, Idir Ghout, Layide Meaude, Pascale Pradat-Diehl, Gaëlle Nelson, Claire Vallat-Azouvi, James Charanton, Philippe Aegerter, Philippe Azouvi

**Affiliations:** ^1^Physical Medicine and Rehabilitation Department, Assistance Publique des Hôpitaux de Paris, Pitie-Salpetriere Hospital, Paris, France; ^2^Physical and Rehabilitation Department, Sorbonne Université GRC18, Paris, France; ^3^Global Brain Health Institute, University of California, San Francisco, San Francisco, CA, United States; ^4^Physical Medicine and Rehabilitation Department, Caen Faculty Hospital, INSERM U1077, France; ^5^Physical Medicine and Rehabilitation Department, Lapeyronie Faculty Hospital, Montpellier, France; ^6^Department of Biostatistics, Assistance Publique des Hôpitaux de Paris, Ambroise Paré Hospital, Boulogne, France; ^7^Unité de Recherche Clinique Paris Ile-de-France Ouest, Ambroise Paré Hospital (APHP), Paris, France; ^8^Laboratoire d'Imagerie Biomedicale INSERM U1146, Paris, France; ^9^Regional Reference Center for Brain Injury in the Parisian Area, Paris, France; ^10^Laboratoire de Recherches Cliniques et en Santé publique sur les Handicaps Psychiques, Cognitifs et Moteurs (HANDIReSP, EA4047), Université de Versailles Saint-Quentin, Montigny-Le-Bretonneux, France; ^11^Physical Medicine and Rehabilitation Department, Assistance Publique des Hôpitaux de Paris, Raymond-Poincaré Faculty Hospital, Garches, France

**Keywords:** traumatic brain injury, litigation, compensation, disability, Paris-TBI, follow-up, lawsuit

## Abstract

**Purpose:** People with traumatic brain injury are frequently involved in a litigation because another person was at fault for causing the accident. A compensation amount will often be settled to compensate the victim for the past, present, future damages and losses suffered. We report descriptive data about the full and final personal compensation amount and investigated its association with patient's outcomes.

**Methods:** We used a longitudinal prospective study of severe TBI patients injured in 2005–2007 (PariS-TBI). Questions regarding involvement in a litigation were asked concurrently with 4 and 8-year outcomes.

**Results:** Among 160 participants assessed 4 and/or 8 years post-injury, a total of 67 persons declared being involved in a litigation, among which 38 people reported a compensation amount of a mean €292,653 (standard deviation = 436,334; interquartile 25–50–75 = 37,000–100,000–500,000; minimum = 1,500-maximum = 2,000,000). A higher compensation amount was associated with more severe disability and cognitive impairment in patients, and with more informal care time provided by caregivers. However, no significant association related to patient's gender, age, years of education, motor/balance impairment, return to work status, mood and related to caregiver's subjective burden was found.

**Conclusion:** Financial compensation was related to victims' long-term severity of impairment, although some extreme cases with severe disability were granted very poor compensation.

## Introduction

Traumatic brain injury (TBI) occurs at a high incidence with more than 50 million people sustaining a TBI each year worldwide (1). Related to this, people with TBI are frequently involved in a litigation with claim compensation proceedings because another person was partly or wholly at fault for causing the injury, in particular in the context of road traffic accidents. While lawsuits after TBI are frequent, research exploring how litigation and long-term TBI outcomes relate to each other is, on the contrary, quite rare (2).

Litigants who sustained a TBI might hire a private attorney or conduct direct negotiation and settlements with insurance companies that both often recourse to some clinical expertise. Together, they will document the personal injury case in order that a monetary value is settled for the past, present and future damages and losses suffered. The health state and the social economic position in which the victim would have been if the accident had not occurred are considered to determine the personal compensation amount (and life rents when applicable) (3). Yet, settling such a monetary value entails numerous levels of complexities and final compensation amounts settled by the court or during out-of-court negotiations vary widely, at least within the French medico-legal context.

There is a lack of knowledge in the literature about personal compensation amounts after TBI, and reports exploring their relationship with TBI long-term outcomes and needs of patients and families are rare. The litigation process in France is entirely separated from clinical care with distinct physicians for both, and information about litigation usually comes from family associations. People sustaining a TBI are not all adequately and equally informed about litigation procedures in France, and some of them do not seek support or advice from a lawyer. Litigation is also a challenging process because cognitive-behavioral impairment requires complex and in-depth evaluations to assess TBI full and long-term impact. As opposed to physical, orthopedic, and motor deficiencies that have often straightforward consequences, the relation of cognitive-behavioral impairment to the disability situation of the victim (in terms of functional outcome in daily life) remains varied and complex to assess.

TBI causes major long-lasting neurological impairments and the related disability is associated with a huge burden for patients and families (1). While litigation process is often a long and stressful experience for TBI litigants (4), it also brings financial compensation that might positively modulate the future life and quality of life of patients and families (5). Thus, investigating whether personal compensation amount is related to the level of disability and needs of the victim seems crucial. We sought here to report descriptive data about the full and final personal compensation amount and long-term patient's outcomes for people with severe TBI who sustained an accident caused by another party. We took the patient's perspective and only investigated the capital sum awarded to the patient by the defendant insurance (i.e., not including the social and medical health expenses paid by the defendant insurance to the health care system, and not including the potential annuities that might be additionally paid by the employer to the patient in case of a work-related injury). We used research follow-up data from the PariS-TBI study (i.e., a French longitudinal inception cohort study of patients with severe TBI, as opposed to a medico-legal dataset). In a previous work using the PariS-TBI study data (4), we showed that those patients involved in a litigation procedure within French jurisdiction compensation scheme had a worse prognosis 4 years after the accident than non-litigant patients in terms of autonomy, participation and psychiatric function. The present extension study on litigation data in PariS-TBI study aims to further investigate the relationship between final personal compensation amount and patient's outcomes after the court verdict or end of negotiations.

## Methods

This study is part of the larger PariS-TBI study undertaken in 2005 in the Parisian area. PariS-TBI is an ongoing inception population-based cohort of individuals with a severe TBI, for which prospective collection included pre-traumatic and early data, and follow-up assessments 1, 4, and 8 years post-injury. Individuals aged 15 or more who had sustained a severe TBI (initial Glasgow Coma Scale score ≤ 8) were consecutively recruited by mobile emergency services over a 22-months period, and assessed in acute care. A total of 504 patients were included (76% men, mean age 42 years). Causes of injury were road traffic accident for 266 (53%), accidental falls for 116 (23%), non-accidental falls for 67 (13%), aggression for 25 (5%), and unknown for 30 (6%). Acute care mortality was 49%, and 134 followed by 147 and 86 survivors were followed-up at 1, 4, and 8 years post-injury, respectively. The 4- and 8-years assessment covered a broad range of impairments, activities, and participation, including questions about the litigation procedure in the form of a face-to-face interview carried out by a neuropsychologist in participant's home. No financial compensation was given to volunteering patients and caregivers. The detailed methodology, longitudinal results and potential biases related to lost-to-follow-up patients have been previously reported (6–13).

Socio-demographic data (age, gender, years of education) and initial severity data [Glasgow Coma Scale score and Injury Severity Score (ISS) (14)] were included. The ISS is an anatomical scoring system that screen for multiple injuries divided into six body regions (head, face, chest, abdomen, extremities including pelvis, and external). In each of these body regions, the severity of the respective injury is assessed on a six-point ordinal scale called the Abbreviated Injury Scale (AIS) and the total ISS score is obtained from the three most severely injured regions that are squared and summed. Patient's assessments at 4 and 8 years included: the Glasgow Outcome Scale-Extended (GOS-E) (15), which covers seven main areas (consciousness, independence at home, independence outside the home, work, social and leisure activities, family and friends, return to normal life) and provides an ordinal classification of disability in eight categories, ranging from death to upper good recovery; the working status (return to work); the Barthel Index which assesses functional independence and mobility in activities of daily living (16); the motor and balance impairment was assessed through a dichotomized score (motor and/or balance disorders, or not); the DysEXecutive questionnaire (DEX) (17) which measures occurrence of cognitive, behavioral, and emotional changes as a result of impairment of executive functions completed by the primary caregiver of the patient; the Hospital Anxiety and Depression scale (HAD) (18). Caregiver's assessments at 4 and 8 years included: the average informal care time provided to the patient per day (i.e., time dedicated to basic and instrumental activities of daily life and supervision) assessed thanks to the Resource Utilization in Dementia battery (RUD) (19); the measure of level of perceived burden thanks to the Zarit Burden Inventory (ZBI) which enable grading the severity of burden experienced by the caregiver into four groups (mild, mild to moderate, moderate to severe, and severe burden) (20). In addition, patients and their relatives were asked whether they were involved in a litigation procedure (i.e., victim of an injury caused by another responsible party and involved in a lawsuit compensation claim related to this injury). When the litigation procedure was settled, patients were asked to report the full and final compensation amount (rounded, in euros) offered and agreed with the opposing party.

Statistical analyses were performed using STATA v14 and R 2.12.0. Comparisons between groups (men vs. women; those people who returned to work vs. those who did not; those with motor and/or balance impairment vs. those without this impairment) were performed using Wilcoxon or Mann–Whitney tests. Spearman correlation tests were used to evaluate the association between the compensation amount and patients' and caregivers' sociodemographic and clinical data.

In accordance with French legislation, patients and their relatives were informed about the inclusion in the database and informed written consent was obtained before each assessment. Approval from Commissions that enforce research database legislation in France and approval from the Ethical Committee (Comité de Protection des Personnes XI) was obtained before each assessment. The study was registered in ClinicalTrials.gov in August 2011 (identifier: NCT01437683).

## Results

**Figure 1:**
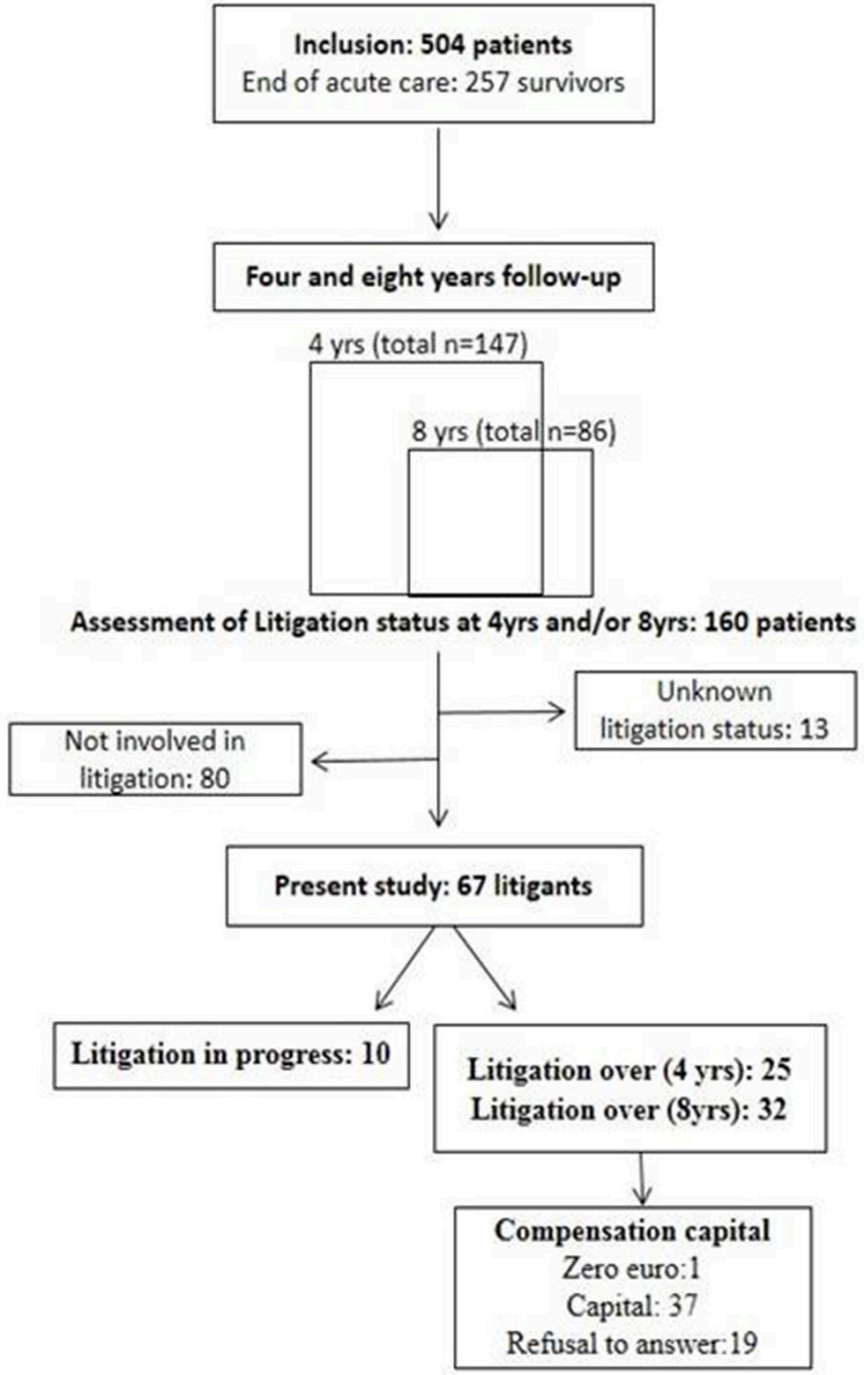
Flow chart depicting PariS-TBI cohort and the litigation study.

Among 160 participants assessed 4 and/or 8 years post-injury, 67 persons declared being involved in a litigation. The litigation was over after 4 and 8 years for 25 and 32 people, respectively, while still in progress for 10 of them, and a total of 38 people (65%) agreed to report the final compensation amount awarded (Figure 1). These 38 litigants included 29 men and 9 women, and the cause of the TBI was road traffic accident in 31 cases, physical aggression in 2 cases and unknown in 5 cases. Regarding litigants with a report of a compensation amount (*n* = 38) vs. those litigants without a report of a compensation amount (*n* = 29), there was no significant differences in socio-demographic and clinical scores (all *p* > 0.05), except for the Zarit Burden Inventory which was lower in litigants with a report of compensation amount (ZBI = 22.9 vs. ZBI = 33.3; *p* = 0.04). Among them, 34 were aged below 70 years of age, including 23 people who were not working and 11 who had a professional activity. Patients' and caregivers' characteristics and scores are presented in Table 1.

**Table 1:** Demographic characteristics and TBI outcomes.

**Variables**	**Mean (standard deviation; minimum – maximum) or count (%)**
**DEMOGRAPHIC CHARACTERISTICS**	
Age (eight years post-injury)	37.5 (15.3; 20.3–80.3)
Years of education	12.7 (3.3; 9–22)
**INJURY SEVERITY**	
Initial Glasgow Coma Scale (min–max = 3–15)	5.6 (1.8; 3–8)
Injury Severity Score (min–max = 0–75)	31.9 (9.9; 14–50)
**PATIENTS AND CAREGIVER FOLLOW-UP OUTCOMES**	
Glasgow Outcome Scale-Extended (1 = death to 8 = upper good recovery)	5.4 (1.3; 3–8)
DysEXecutive questionnaire (min-max = 0–80)	24.9 (15.1; 0–71)
Motor and/or balance deficiency (yes)	15/38 (39%)
Hamilton Anxiety and Depression scale (min-max = 0–42)	12.6 (8.5; 0–29)
Barthel index (min-max = 0–100)	97.4 (7.2; 70–100)
Ressource Utilization in Dementia scale (min-max = 0–24)	6.5 (8.5; 0–24)
Zarit Burden Inventory (min-max = 0–88)	22.9 (16.2; 0–59)

The final settlement amount was zero euro for 1 person and a mean €292,653 (standard deviation = 436,334; interquartile 25–50–75% = 37,000–100,000–500,000; minimum = 1,500–maximum = 2,000,000) for the others. Among the most severe patients (with GOS-E scores 3 and 4), 4 patients had a low amount below 88,000 euros. There was a non-significant tendency (*p* = 0.2) for higher compensation amount in men with a mean €343,166 (standard deviation = 490,184; median = 100,000; minimum–maximum = 5,000–2,000,000) as opposed to a mean €135,500 (standard deviation = 95,298; median = 115,000; minimum = 1,500–maximum = 300,000) in women. In those people below age 70 eight years post-injury (*n* = 34), there was a non-significant tendency (*p* = 0.08) for higher compensation amount awarded in those who had not returned to work (*n* = 23) with a mean €407,094 (median = 2,00,000; standard deviation = 516,331; minimum = 1,500–maximum = 2,000,000) vs. a mean €117.727 (median = 80,000; standard deviation = 134,498; minimum = 5,000–maximum = 420,000) in those who had returned to work (*n* = 11). There was no difference in compensation amount (*p* = 0.9) in those with motor and/or balance impairment with a mean €293,333 (standard deviation = 415,464; minimum–maximum = 63,257–523,409) as opposed to mean €292,189 (standard deviation = 459,679; minimum–maximum = 88,378–495,999) in those without such deficiency.

**Figure 2:**
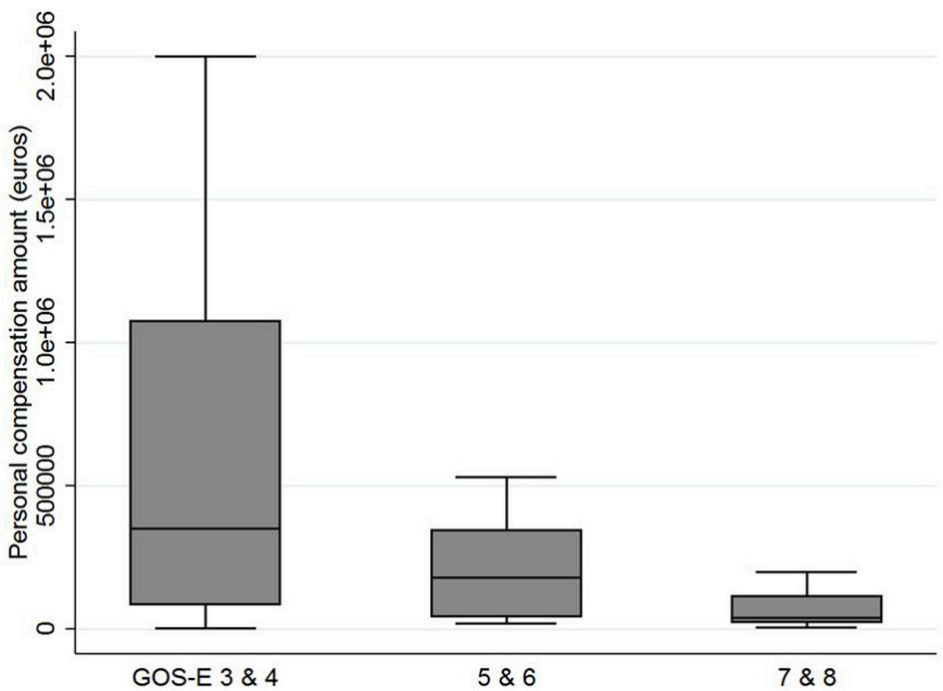
Final personal compensation amount offered to litigants as a function of the Glasgow Outcome Score-Extended. Disability level, number of patients (*n*) and age (mean, range[]) per category: GOS-E 3 and 4 = severe (*n* = 10; 43.6 [22-80]); 5 and 6 = moderate (*n* = 16; 39.2 [21-71]); 7 and 8 = light (*n* = 11; 29.7 [20-48]).

Regarding socio-demographic data, no correlation was found between compensation amount and age or years of education (*p* = 0.9 and *p* = 0.3, respectively). Regarding initial severity, no correlation was found between compensation amount and Glasgow Coma Score or ISS (*p* = 0.4 and *p* = 0.7, respectively). Regarding patients' 4 and 8 years outcomes (depending on when the verdict happened): a higher compensation amount was associated with a more severe disability as assessed with the GOS-E (Spearman's rho = 0.4, *p* = 0.01 as illustrated on Figure 2); a higher compensation amount was associated with more severe cognitive impairment on the DEX (Spearman's rho = 0.4, *p* = 0.02); no such association was found with the Barthel index score (*p* = 0.1) nor for anxiety and mood (HAD, *p* = 0.2); in caregivers, a higher compensation amount was associated with more informal care time devoted to the patient (rho = 0.66, *p* = 0.01) but not with a higher subjective burden (ZBI) (*p* = 0.07).

## Discussion

TBI lawsuit settlements result in a secure financial capital in the form of a personal compensation amount offered to the victims who sustained an accident caused by another party. Personal compensation amount were variable, and a higher compensation was found associated with more severe levels of global handicap, of executive dysfunction and of informal care time, but no significant association was found with patients' age, years of education, initial severity scores (including the ISS which takes into account extracranial injuries), motor-balance impairment, return to work, mood nor with subjective burden in caregivers. These figures and associations are discussed in light with the possible mediating factors that were unmeasured given the exploratory nature of the present study.

In France, negotiations and medico-legal expertise around personal compensation use various abacuses to support objectivity in the medico-legal proceedings, assessing a systematic range of heads of damages (including pecuniary and non-pecuniary sectors, temporary and permanent damages, and damages to direct and indirect victims) (21). We report here the full and final personal compensation amounts granted to individuals (i.e., not including the social and medical health expenses paid by the defendant insurance to the health care system).We found a substantial variability in personal compensation amounts ranging from €1,500 to €2,000,000 (i.e., $1,709 to $2,278,982). Original research and reports on financial compensations in TBI victims are lacking in France and elsewhere, making explanations and comparisons in the field challenging. Jou's group is one of the few to use data about compensations that perpetrators were ordered to pay in court verdicts in 2013 in new Taiwan dollars (22, 23). Yet, the authors did not focus on verdict compensation amounts but rather used them to assess the amount that road accident perpetrators were willing to pay to compensate their victims. Using hypothetical scenarios (contingent valuation method) they found that perpetrators were willing to pay more consolation compensation with increased injury severity. Data about compensations amount in the community stem from law firms freely available on the web providing ranges of values or case results: for instance a range from £1,940 to £11,200 (i.e., $2,452–14,154) in mild head injury and a range from £247,280 to £354,260 in major head injury (i.e., $312,498–447,694) can be found in the United Kingdom (24); for instance an isolated case reported very high monetary values of $17.4 million (granted to a 27 years old man injured by a truck in 2012) (25) or a set of case results reported a range from $100,000 (mild concussion) to $26 million (permanent brain damage) in the United States (26). Yet one must keep in mind that these data are displayed by the law firms to inform the general population and also for the firms marketing purposes, but not for scientific research.

That higher compensation amounts were positively correlated with more severe handicap (as assessed by the routine score GOS-E) seems reinsuring: it suggests that finances granted through litigation are adapted to the level of recovery and needs of victims who often experience a socio-economic precarity due to TBI (because of the loss of a job and a regular income for instance) (1). While we did not find research documenting such a congruent relation between global disability and financial settlement, this result aligns with legal information freely available in the community (27). As the prolonged process of litigation against a defending party might have a negative impact on patient's recovery and community reentry (2, 4, 28), it is necessary to show positive benefits in the form of financial award to confirm that victims should proactively pursue lawsuits. For instance, financial compensation was found to have a protective effect against late mortality following rehabilitation for severe TBI (through interactions with rehabilitation service variables), suggesting that wider access to compensation (and rehabilitation) might further improve life expectancy in TBI (5).

The correlation with cognitive disorders is interesting as cognitive impairment is a core factor impacting negatively patients' quality of life, autonomy, community reentry and economic status (1, 6, 7). While cognitive-behavioral sequelae might be sometimes overlooked as a consequence of unawareness of invisible impairment they were associated here with a higher compensation amount while motor-balance impairment was not. The correlation between compensation amount and caregiving needs (as assessed by the number of informal caregiving hours devoted to the patient) seems also an interesting finding as informal caregivers are often called “ricochetting victims” (4, 5). Informal caregivers are known to bare the burden of care in TBI and the economic valuation of their informal work in litigation represent a significant financial head of damage (1, 5).

Surprisingly though, the financial compensation did not significantly differed among those litigants who worked again vs. those who did not. This might appear counterintuitive as compensation is being capitalized on years lived with a disability and typically on loss of earnings and career chances related to the head of damages “return to work” (3, 21). It might be that our sample size was too small to ascertain this tendency statistically. Also, a younger age might not be associated with a higher amount maybe because capitalization of the career loss could not be achieved in patients who were students at the time of the accident. The non-significant relation between compensation amount and initial severity of the injury might point out a relative small impact of the initial damages as opposed to appraisal of long-term sequelae and social and economic heads of damages.

The findings of the present study should be interpreted with caution though as many parameters might play a role in the compensation process. Apart from the severity of the injury and of the associated impairments, a set of other unmeasured factors here might account for court outcomes and compensation amounts. For instance, pre-injury chronic conditions classically assessed in epidemiology with the Charlson comorbidity score were not measured here. Yet, premorbid impairments could contribute along with the consequences of the TBI to the global disability of people and to the various impairments and informal care needs measured here, while logically not resulting at the same time in a higher financial compensation. In that case, forensic evaluation should adequately disentangle medical and social economic consequences uniquely related to TBI from those related to prior conditions and comorbidity. We do not know the cause about poor compensation in some individuals of the sample and might only make assumptions based on care experience. Sometimes the expertise process might result in a rather unfair settlement (with extreme cases where people with most severe disability were granted very poor financial compensation amounts for life). In the case of an unfair compensation system, health professionals would be strongly encouraged to refer litigant patients to specialized attorneys or consulting physicians to assist them in this complex process. Yet, isolated TBI vs. TBI associated with multiple extra-cranial injuries might also account for a great inter-individual variability of compensation amounts. Other categories of loss that were not capture in the present study, such as esthetic impairment, loss of sexual function, loss of the prospect of founding a family, pain, expenses of accommodation, and vehicle conversion, loss of opportunity regarding education could result in ascertainment bias of our results. These unmeasured factors might account for the extreme cases we observe where people with most severe disability were granted very poor financial compensation amounts for life. In addition, other external factors such as the attorney's skills, and insurance expert's experience (or reversely, malpractice leading to underreporting of impairment), the court jurisdiction specificities, the solvability of the opposing party, the influence of personality of patients during legal proceedings might also negatively (or positively) influence the settlement of the final compensation outcome and account for these disparities in amounts and consequential social inequalities. A final issue is that a number of patients were lost to follow-up, as in most long-term follow-up research. As previously reported, included patients one and four-year post injury did not significantly differ from lost-to-follow up patients in terms of injury severity, However, social and demographic factors such as unemployment before the injury or pre-injury alcohol abuse were significantly associated with loss to follow-up (8). This may be a potential source of bias.

As a whole, caution is needed because of the small size of the sample and further research is warranted to continue investigating these compensation outcomes and associations in other international settings and larger datasets. Future study should examine in detail the full list of losses and outcomes related to predicted life path. Inclusion of financial life rents and all other types of medical expenses and reimbursements of acute and long-term care would also provide a better overview of the compensation situation. The main limitations of the present study lie in the small sample size that does not permit to compute multivariate model analyses, and in the limited list of heads of damage available here, including identifying from the start isolated TBI vs. TBI associated with multiple extra-cranial injuries. A strength however lies in the nature of PariS-TBI study that was not meant to gather clinicolegal data but rather provides a neutral independent setting of examination in the context of litigation.

## Author Contributions

EB had full access to all the data in the study and takes responsibility for the integrity of the data and the accuracy of the data analysis. EB, CJ, AR, and PA: concept and design; All authors: acquisition, analysis, or interpretation of data; EB, CJ, AR, and PA: drafting of the manuscript; All authors: critical revision of the manuscript for important intellectual content; EB, IG, and CJ: statistical analysis; CJ: obtained funding; EB, CJ, PA, IG, PA, and LM: administrative, technical, or material support; PA: supervision.

### Conflict of Interest Statement

The authors declare that the research was conducted in the absence of any commercial or financial relationships that could be construed as a potential conflict of interest.
